# Accelerated Vascular Aging in CuZnSOD-Deficient Mice: Impact on EPC Function and Reparative Neovascularization

**DOI:** 10.1371/journal.pone.0023308

**Published:** 2011-08-12

**Authors:** Jessika Groleau, Sylvie Dussault, Julie Turgeon, Paola Haddad, Alain Rivard

**Affiliations:** Department of Cardiovascular Research, Centre Hospitalier de l'Université de Montréal, Montréal, Québec, Canada; University of Padova, Italy

## Abstract

**Objective:**

Aging is associated with increased oxidative stress levels and impaired neovascularization following ischemia. CuZnSOD has an important role to limit oxidative stress in the vasculature. Here we investigated the role of CuZnSOD for the modulation of ischemia-induced neovascularisation during aging.

**Methods and Results:**

Hindlimb ischemia was surgically induced in young (2- month-old) or older (8-month-old) wild type (WT) and CuZnSOD^−/−^ mice. We found that blood flow recovery after ischemia and vascular density in ischemic muscles were significantly reduced in older compared to young WT mice. Both in young and older mice, CuZnSOD deficiency led to a further reduction of neovascularization. Accordingly, the resulting neovascularisation potential in a young CuZnSOD^−/−^ mouse was similar to that of an older WT mouse. Oxidative stress levels were also increased to similar levels in the ischemic muscles of young CuZnSOD^−/−^ and older WT mice. To identify potential mechanisms involved, we investigated the effect of aging and CuZnSOD deficiency on the number and the function of endothelial progenitor cells (EPCs). Both aging and CuZnSOD deficiency were associated with reduced number of bone marrow and peripheral EPCs. The effect of moderate aging alone on specific functional activities of EPCs (migration, integration into tubules) was modest. However, CuZnSOD deficiency was associated with severe age-dependent defects in EPC functional activities.

**Conclusions:**

CuZnSOD deficiency is associated with accelerated vascular aging and impaired ischemia-induced neovascularization. Our results suggest that in the context of aging, CuZnSOD has an essential role to protect against excessive oxidative stress in ischemic tissues and preserve the function of EPCs.

## Introduction

In patients with cardiovascular diseases, the capacity of the organism to develop new blood vessels (neovascularization) constitutes an important adaptive mechanism against ischemia [1]. Recent studies suggest that postnatal neovascularization relies not exclusively on the sprouting of mature endothelial cells in pre-existing vessels (angiogenesis), but also involves the contribution of bone marrow-derived circulating endothelial progenitor cells (EPCs) [2], [3]. It has been demonstrated that circulating EPCs in adults can home to ischemic tissues and contribute to the formation of new blood vessels [4].

Advanced age is a major risk factor for coronary and peripheral artery disease. In addition, one of the consequences of aging is a decline in the ability of the organism to respond to different stresses, including ischemia. For instance, advanced age is associated with a defect in neovessel formation following arterial occlusion in different animal models [5], [6]. Moreover, the number and/or the functional activities of EPCs have been shown to be impaired by aging both in animals and in humans [7], [8], [9], [10]. However, the precise mechanisms involved in the modulation of neovascularisation and EPC function by aging remain to be determined.

A loss of the adaptive response to oxidative stress with the passage of time is one of the major characteristic of aging [11], [12], [13]. Oxidative stress level in the vasculature is the result of a balance between the rate of ROS formation and the rate of ROS removal by endogenous antioxidant enzymes such as superoxide dismutases (SODs). The predominant isoform of SOD within blood vessels is copper-zinc SOD (CuZnSOD; SOD1), accounting for 50% to 80% of total SOD activity [14]. CuZnSOD is located within the cytosol as well as in the nucleus, and is thought to be expressed in all mammalian cells. In heterozygous CuZnSOD-deficient mice, increases in superoxide levels and impaired vasodilatation have been documented in old but not in young animals [15]. However, the effect of aging on vascular function has not been investigated in homozygous CuZnSOD-deficient (CuZnSOD^−/−^) mice. Moreover, the functional importance of CuZnSOD for the age-dependent modulation of ischemia-induced neovascularization is currently unknown.

Here we used a mouse model of hindlimb ischemia to study the effect of CuZnSOD deficiency on oxidative stress levels and reparative ischemia-induced neovascularization in the context of aging. We found that CuZnSOD-deficient mice exhibit accelerated vascular aging and impaired neovascularization in response to ischemia. We also demonstrate that CuZnSOD has an essential role to maintain the functional activities of EPCs in older animals.

## Methods

### Experimental Animals

Mice used for this study were derived from breeding pairs of heterozygous CuZnSOD-deficient (B6;129S7-SOD1^tm1Leb^/J) mice obtained from Jackson Laboratory (Bar Harbor, Maine). Four groups of mice were studied: young (2-month-old) and older (8-month-old) homozygous CuZnSOD-deficient (CuZnSOD^−/−^) mice were compared to young and older wild-type (CuZnSOD^+/+^) littermates. Mice were maintained in 12 hours light-dark cycle and fed *ad libitum*. Genotyping of each mouse was assessed by polymerase chain reaction of DNA isolated from tail biopsy samples as described on the Jackson laboratory Web site.

### Murine ischemic hindlimb model

The protocol (N10011ARs) was approved by the Comité Institutionnel de Protection des Animaux (CIPA) of the Centre Hospitalier de l'Université de Montréal (CHUM). Unilateral hindlimb ischemia was surgically induced in mice as previously described [16]. Briefly, the animals were anesthetized with 2% isoflurane, after which an incision was performed in the skin overlying the middle portion of the left hindlimb. After ligation of the proximal end of the femoral artery, the distal portion of the saphenous artery was ligated, and the artery and all side branches were dissected free and excised. The skin was closed with a prolene monofilament (6-0) (Johnson & Johnson, ON, Canada).

### Monitoring of hindlimb blood flow

Hindlimb perfusion was measured with a Laser Doppler Perfusion Imager (LDPI) system (Moor Instruments Ltd., Axminster, UK). After anesthesia with a ketamine-midazolam solution (100 mg/kg-5 mg/kg, intraperitoneally), consecutive measurements were obtained after scanning of the same region of interest (leg and foot) with the LDPI. The perfusion signal was split into six different intervals, each displayed in a separate color. Low or no perfusion was displayed in dark blue, whereas the highest perfusion interval was displayed in red. Color photographs were recorded and analyses were performed by calculating the average perfusion of the ischemic and non-ischemic hindlimb. To account for variables such as ambient light and temperature, the results are expressed as the ratio of perfusion in the left (ischemic) vs. right (non-ischemic) hindlimb. The mice were killed at predetermined arbitrary time points after surgery with an overdose of sodium pentobarbital.

### Tissue preparation and immunochemistry

For immunohistochemistry, whole ischemic hindlimbs were immediately fixed in tissue-fix overnight. After bones had been carefully removed, 3-µm thick tissue transverse sections of the hindlimbs were cut at the level of the gastrocnemius muscle and paraffin-embedded so that the whole leg could be analyzed on each section. Identification of endothelial cells was performed by immunostaining for platelet endothelial cells adhesion molecule-1 (PECAM-1 or CD31) with a rat monoclonal antibody directed against mouse CD31 (Pharmigen, San Diego, CA). Capillaries, identified by positive staining of CD31 and appropriate morphology, were counted by a single observer blinded to the treatment regimen under a 200× magnification to determine the capillary density (number of capillaries per mm^2^). Serial sections were cut at three different levels, and representative fields were analyzed by counting the number of capillaries in each field. Arterioles were identified using a Modified Verhoeff Van Gieson Elastic Stain Kit (Sigma, St. Louis, MO). Serial sections were cut at three different levels, and positive vessels identified by the presence of a continuous internal elastic laminae and muscle spindle were analyzed for the entire section under a 100× magnification. To characterize premature vascular senescence, goat polyclonal antibodies against mouse p16 and p53 (Santa Cruz Biotechnology, Santa Cruz, CA) were used. To investigate local oxidative stress levels in ischemic muscles, an antibody against nitrotyrosine (Upstate, Lake Placid, NY) was used. Nitrotyrosine immunostaining is a reflection of peroxinitrite produced locally in tissues. Intensities of fluorescence were measured and analyzed using computer-based analysis (Metamorph) with the same threshold for all sections under a 200× magnification. The specificity of the test was confirmed by pre-incubating the antibody with 10 mM nitrotyrosine (data not shown). To evaluate superoxide production in ischemic muscles, dihydroethidium (DHE) fluorescence labeling was performed. After bones had been carefully removed at the level of the gastrocnemius muscle, ischemic muscles were put in 25% sucrose solution for 10 minutes. Muscles were frozen in eppendorf tubes at −80°C for 24 hours, mold in OCT and kept at −20°C. 3 µm frozen sections were made at three different levels in the ischemic muscles. Sections were labeled with 10 µM DHE (Calbiochem, San Diego, CA) for 30 minutes. Intensities of fluorescence were measured and analyzed using a computer-based software (Metamorph) with the same threshold for all sections under a 100× magnification. The specificity of the test was confirmed by pre-incubating the section with superoxide dismutase polyethyleneglycol (PEG-SOD) 500 U/ml for 1 hour (data not shown).

### Western blot analysis

Whole-cell protein extracts were obtained after homogenization of muscles from hindlimbs of young and older mice in a lysis buffer containing 50 mM HEPES pH 7.6, 150 mM NaCl, 1 mM EDTA, 25 mM β-glycerophosphate, 1 mM sodium orthovanadate, 1 mM NaF, 0.1% tween 20, 10% glycerol, 1 mM DTT, 1 mM phenylmethylsulfonyl fluoride, 1 µg/mL leupeptin, and 1 µg/mL aprotinin. Protein concentrations were measured according to the Bradford method and equal amount (100 µg) of protein extract was separated in nonreducing 15% polyacrylamide gel and electroblotted on nitrocellulose membranes. The membranes were probed with 1∶500 CuZnSOD antibody (Chemicon, Billerica, MA). Specific proteins were detected by chemiluminescent reaction (GE Healthcare Bio-sciences, Piscataway, NJ). Results are expressed as density values normalized to Ponceau red staining.

### FACS analysis of Circulating Progenitor Cells

Progenitor cells contained in the total viable population derived from the spleen were analysed by FACs (FACSCalibur flow cytometer, Becton Dickenson, Oakville, Ontario, Canada), using fluorescence-coupled antibodies against the following markers: CD34-FITC, VEGFR2 (Flk-1)-PE and CD117 (c-kit)-APC (eBioscience, CA, USA). Corresponding isotype-matched immunoglobulins for the three antibodies gave similar low levels of fluorescence compared to unstained cells confirming that the primary antibody binding is specific and not a result of non-specific Fc receptor binding or other protein interactions. The results are expressed as the percentage of triple-marked cells from the initial population. Cell phenotypes were determined by the analysis of 300 000 events.

### Endothelial Progenitor Cells isolation and characterization (early outgrowth EPCs)

Seven days following ischemia, mononuclear cells were mechanically isolated from the femora, tibiae and humeri by flushing the bone marrow cavities using culture medium. After red blood cell lysis and washing, mononuclear cells were plated on 0.05% fibronectin (Sigma) and cultured in complete Medium 200 containing 20% FBS. After 4 days in culture, non-adherent cells were removed by thorough washing with PBS. Adherent cells were stained with 1,1′-dictadecyl-3,3,3′,3′ tetramethyllindocarbocyanine perchlorate-acetylated low-density lipoprotein (DiI-acLDL, 2.5 µg/ml for 1 hour, Invitrogen, OR, USA) and FITC-labeled lectin BS-1 (Bandeiraea simplicifolia, 10 µg/ml for 1 hour, Sigma). Spindle-shaped cells were observed, and the vast majority of adherent cells (95%) were found to be double-positive for the uptake of DiI-acLDL and binding of FITC-labeled lectin. FACS analyses revealed that 83% of adherent cells express CD45, 57% CD14, 18% CXCR4, 9% CD31 and 8% Sca-1. These cells were shown to migrate in response to VEGF stimulation and were capable of incorporation into a network of tubular-like structures when cocultured with mature endothelial cells. On the basis of these morphological and functional characteristics, these cells were defined as early outgrowth EPCs.

### Detection of intracellular superoxide oxygen radicals in EPCs

Dihydroethidium (DHE) was used to evaluate the presence of superoxide anion oxygen radicals. In the presence of O_2_^−^, DHE is oxidized to ethidium bromide, which binds to DNA. EPCs were plated at a density of 15000/well in a 96-well plate in complete Medium 200, 20% FBS for 24 hours. The fluorescence reaction was carried out by incubating EPCs with DHE (2 µmol/L) for 30 minutes. DHE fluorescence was detected with a fluometer (Fluostar OPTIMA, BMG Labtechnologies, Offenburg, Germany) using a 585 nm filter. The specificity of the test was confirmed using 50 ng/ml TNF-alpha and 500 U/ml PEG-SOD as positive and negative controls, respectively (data not shown).

### Cell migration assay

Cell migration was assessed using a modified Boyden chamber assay. Polyvinylpyrrolidine-free polycarbonate filter Transwell inserts (6.4 mm diameter, 8 µm pores; Costar, Cambridge, MA) were coated with 0.1% gelatin. Inserts were placed in a 24-well plate containing DMEM with 50 ng/ml of VEGF. EPCs (15 000) were added to the upper chamber of the inserts in DMEM, 0.1% fetal bovine serum. Cells were allowed to migrate from the upper to the lower chamber for 6 h at 37°C. Non-migratory cells were removed from the upper chamber by wiping the upper surface with an absorbent tip. Cells that had migrated to the lower side of the transwell insert were fixed for 10 min with 3.7% formaldehyde and stained with hematoxilin. The number of cells that had migrated was counted in six different representative high power (200×) fields per insert (2 inserts/condition). The data are presented as number of migrated cells ± standard error from the mean.

### EPCs adhesion to endothelial cells

A monolayer of human umbilical vein endothelial cells (HUVECs) was prepared 48 hours before the assay by plating 2×10^5^ cells (passage 2–5) in each well of 24 wells plate. HUVECs were pretreated for 12 hours with tumor necrosis factor-α (BD Biosciences, San Diego, CA) (1 ng/ml). Cells were stained with DAPI and fixed with 2% paraformaldehyde. EPCs were labeled with DiI-acLDL and 11 000 EPCs were added to each well and incubated for 3 hours at 37°C. Non-attached cells were gently removed with PBS and adherent EPCs were fixed with 2% paraformaldehyde and counted in three random fields. The data are presented as a ratio of the number of adherent EPCs/HUVECs per field ± standard error of the mean.

### Capillary-like tube formation on Matrigel

EPC (4000) labeled 1 hour with DiI-acLDL were co-plated with HUVECs (14 000) in 96-well plates that had been precoated with 50 µl of growth factor reduced Matrigel Matrix (BD Biosciences) and cultured at 37°C for 6 h with 50 ng/ml of VEGF. Tubular-like structures were photographed and the number of incorporated EPCs in tubules was determined in 6 random fields. A tube was defined as a straight cellular segment connecting two cell masses (nodes). The data are presented as number of incorporated EPCs/tube ± standard error of the mean.

### Statistical Analysis

All results are expressed as mean ± SEM. Statistical significance was evaluated using a one-way ANOVA followed by Newman-Keuls post-hoc test for multiple group comparison. A value of p<0.05 was interpreted to denote statistical significance.

## Results

### Effect of CuZnSOD-Deficiency and Aging on Neovascularization after Ischemia

**Figure 1:**
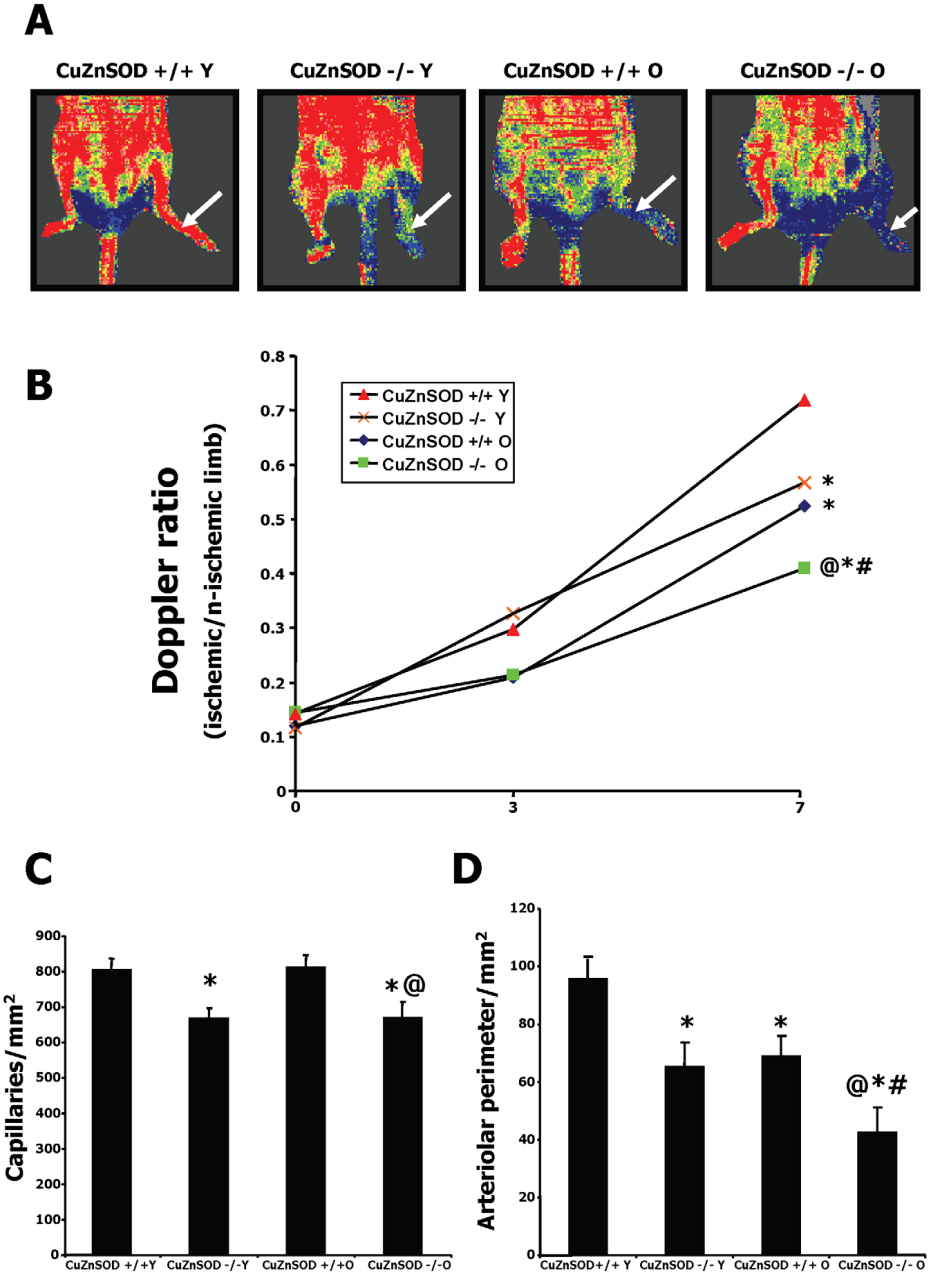
Effect of CuZnSOD deficiency and aging on ischemia-induced neovascularization. A. Representative results of laser Doppler measurements 7 days after hindlimb ischemia in young (Y) and older (O) wild-type (CuZnSOD+/+) and CuZnSOD−/− mice. A color scale illustrates blood flow variations from minimal (dark blue) to maximal (red) values. Arrows indicate ischemic (left) hindlimb. B. Quantification of Laser Doppler perfusion ratios. C–D. Quantification of capillary (C) and arteriolar (D) densities in ischemic muscles harvested at day 7 in the different groups. Data are mean vessels/mm2 ± SEM (n = 7–10/group). *P<0.05 vs. young wild type mice. @P<0.05 vs. old wild type mice. #P<0.05 vs. young CuZnSOD−/− mice.

In control wild type (WT) mice, we found that aging is associated with a significant decrease of CuZnSOD expression in hindlimb muscles (Figure S1). To characterize the role of CuZnSOD-deficiency and aging on neovascularization, blood flow perfusion was evaluated by serial LDPI studies after the induction of mouse hindlimb ischemia (Figure 1A). Immediately after the surgery (day 0), Doppler flow ratios (DFR) between the ischemic and normal hindlimbs were reduced to similar low levels, indicating that the severity of the induced ischemia was similar in all groups (Figure 1B). However, older wild type (WT) mice had a significantly slower rate of blood flow recovery when compared to young mice at day 7 after surgery (0.52±0.03 vs. 0.72±0.03; p<0.01). Both in young and older mice, CuZnSOD deficiency was associated with a further reduction of blood flow recovery. Accordingly, the resulting blood flow perfusion in young CuZnSOD^−/−^ mice was similar to that of older WT mice (DFR 0.57±0.05 vs. 0.52±0.03; p = 0.42). At the microvascular level, capillary density was significantly reduced in CuZnSOD-deficient mice (Figure 1C). Moreover, total arteriolar perimeter in ischemic tissues was reduced both by aging and CuZnSOD deficiency, resulting in values in young CuZnSOD^−/−^ mice that were similar to that of older WT mice (Figure 1D).

### Oxidative Stress Levels and Vascular Aging in Ischemic Tissues

**Figure 2:**
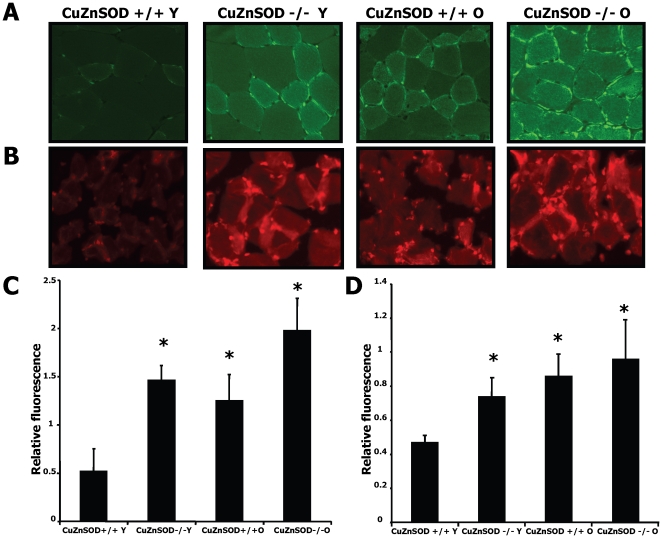
ROS levels in ischemic tissues. Nitrotyrosine (A) and Dihydroethidium (B) immunostaining of ischemic muscles harvested at day 7 after ischemia in young (Y) and older (O) wild-type (CuZnSOD^+/+^) and CuZnSOD^−/−^ mice (n = 10/group). Nitrotyrosine (C) and Dihydroethidium (D) quantification in the different groups is presented as arbitrary fluorescent units. Data are mean ± SEM. *P<0.05 *vs.* young wild type mice.

Because excessive oxidative stress has been associated with impaired angiogenesis in different situations, we compared oxidative stress levels in ischemic muscles of young and older CuZnSOD^−/−^ and WT mice. Figure 2A shows representative results of nitrotyrosine immunostaining, a reflection of peroxinitrite produced locally in tissues. Figure 2B shows results for DHE, an indicator of superoxide levels. Quantification of relative fluorescences (2C and 2D) demonstrates that both markers of oxidative stress were significantly increased by aging and CuZnSOD deficiency. Young CuZnSOD-deficient mice had oxidative stress levels in ischemic tissues that were similar to that of older WT mice, and the highest oxidative stress levels were seen in old CuZnSOD-deficient mice. Interestingly, increased oxidative stress levels in ischemic tissues of CuZnSOD-deficient mice were associated with accelerated vascular aging, as demonstrated by higher expression of stress-induced senescence markers p53 and p16 in capillaries (**Figure S2**).

### Effect of CuZnSOD-Deficiency and Aging on EPC Levels

**Figure 3:**
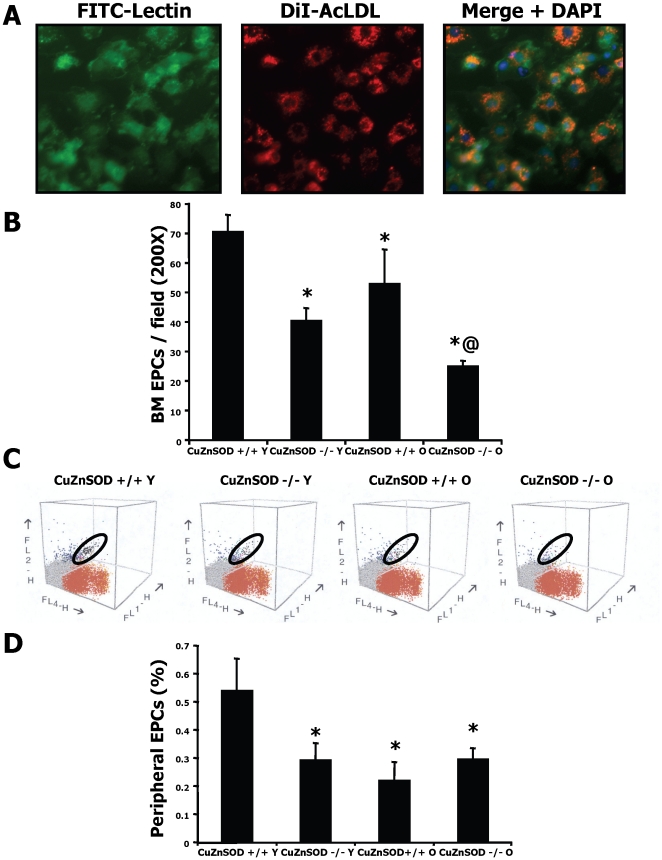
Effect of CuZnSOD deficiency and aging on EPC number. A. EPCs were identified using DiI-acLDL, lectin-FITC and DAPI stains. Cells positive for all 3 dyes were counted as EPCs. B. EPCs isolated from the bone marrow were quantified at day 7 after hindlimb ischemia. C. Circulating endothelial progenitor cells derived from the spleen were analyzed by FACS (300 000 events) using the surface markers CD34 (FL1-H axis), VEGFR2 (FL2-H axis) and CD117 (FL4-H axis). Triple-marked cells are indicated in the black oval and representative examples are shown in the different groups. D. The percentage of triple-marked cells from the initial cell population was quantified (n = 8 mice/group). Data are mean ± SEM. *P<0.05 *vs.* young wild type mice. @*P*<0.05 *vs.* old wild type mice.

To identify potential mechanisms involved in the detrimental effect of aging and CuZnSOD deficiency on ischemia-induced neovascularization, we quantified the number of EPCs in the different animal groups at day 7 after hindlimb ischemia. Early outgrowth bone marrow EPCs were isolated in culture and shown to endocytose acLDL and bind BS-1 lectin (Figure 3A). We found that the number of bone marrow EPCs is reduced both by aging and CuZnSOD deficiency (Figure 3B). Young CuZnSOD-deficient mice had bone marrow EPC levels that were similar to that of older WT mice, and the lowest EPC levels were found in old CuZnSOD-deficient mice. The percentage of circulating progenitor cells contained in the total viable cell population derived from the spleen was also measured by FACS analysis at day 7 after ischemia using the cell markers CD34, VEGFR2 and CD117 (Figure 3C). Similarly to EPCs isolated in culture, the number of peripheral circulating progenitor cells was significantly reduced by aging and CuZnSOD deficiency (Figure 3D). The level of EPCs in young CuZnSOD-deficient mice was similar to that of older WT mice.

### Oxidative Stress and Functional Activities of EPCs

**Figure 4:**
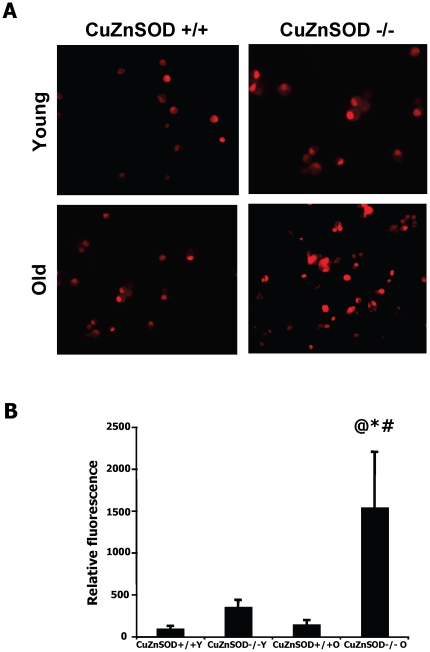
Superoxide levels in EPCs. A. Superoxides were detected using dihydroethidium (DHE) immunostaining. The analyses were performed on EPCs harvested from the bone marrow of young or older wild type (CuZnSOD^+/+^) and CuZnSOD^−/−^ mice at day 7 after hindlimb ischemia (n = 5/group). B. Quantification was performed with a fluorometer and is presented as arbitrary fluorescent units. Data are mean ± SEM. *P<0.05 *vs.* young wild type EPCs. @*P*<0.05 *vs.* old wild type EPCs. #*P*<0.05 *vs.* young CuZnSOD^−/−^ EPCs.

**Figure 5:**
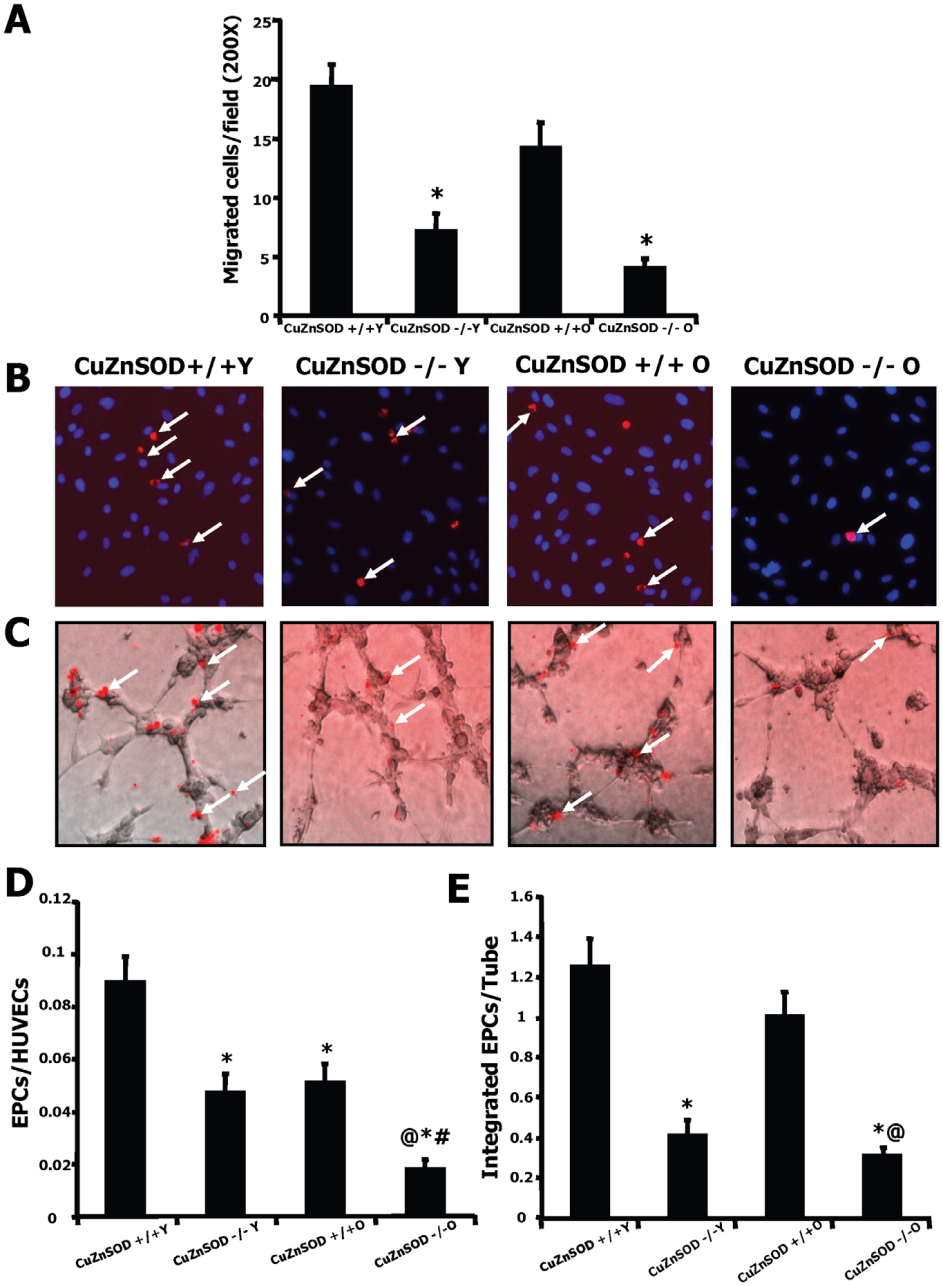
Effect of CuZnSOD deficiency and aging on EPC angiogenic activities. EPCs were isolated from the bone marrow of young or older wild type (CuZnSOD^+/+^) and CuZnSOD^−/−^ mice. VEGF-induced migration (A) was assessed using a modified Boyden chamber assay. To assess EPC adhesion (B–D), EPCs were labeled with a DiI fluorescent marker (red) and allowed to adhere to a monolayer of TNF-α-stimulated HUVECs (blue, nuclear stain DAPI). To assess integration into tubular-like structures (C–E), fluorescent-labeled EPCs (red) were coplated with HUVECs (transparent) to form tubular-like structures on Matrigel. EPCs from 5–7 different mice/group were tested. Data are mean ± SEM. *P<0.05 *vs.* young wild type EPCs. @*P*<0.05 *vs.* old wild type EPCs. #*P*<0.05 *vs.* young CuZnSOD^−/−^ EPCs.

Superoxide generation was measured in EPCs isolated from the bone marrow using DHE immunostaining. A small non-significant increase of superoxide generation was seen in EPCs isolated from older vs. young wild-type animals. However, CuZnSOD deficiency was associated with a very important increase of EPC superoxide levels in older animals (Figure 4A and 4B). We also demonstrate that this increased oxidative stress is associated with an important impairment of EPC functional activities (Figure 5). CuZnSOD-deficiency in EPCs was associated with several characteristics of aging including reduction in VEGF-induced migration (Figure 5A), impairment in the ability to adhere to a HUVEC monolayer activated with TNF-α (Figure 5B and 5D), and reduction in the capacity of EPCs to integrate into tubular-like structures in coculture with HUVECs (Figure 5C and 5E). Although the effect of aging alone on EPC function was often modest, CuZnSOD deficiency was associated with severe age-dependent defects.

## Discussion

To our knowledge, the present study is the first one to investigate the role of CuZnSOD for postnatal ischemia-induced neovascularization in the context of aging. Since oxidative stress is thought to contribute to the pathophysiology of aging [12], and because of the important role of CuZnSOD to limit increases in superoxide levels in different tissues, we hypothesized that CuZnSOD deficiency would accelerate vascular aging and compromise neovascularization in response to ischemia. Previous studies have indicated that CuZnSOD deficiency leads to physiological defects that resemble aging. For example, CuZnSOD^−/−^ mice have been shown to exhibit acceleration of several age-related diseases including skeletal muscle atrophy [17], hepatocarcinogenesis [18], retinal degeneration [19], [20] and hearing loss [21]. In the cardiovascular system, young heterozygous CuZnSOD-deficient mice do not show any significant alteration of endothelial function [15] or postischemic myocardial contractile function [22]. However, impaired endothelial-dependant vasodilatation has been demonstrated in older heterozygous CuZnSOD^+/−^ mice [15], and in young homozygous CuZnSOD^−/−^ mice [14]. Moreover, we have recently shown that homozygous CuZnSOD deficiency in young mice leads to increased oxidative stress levels and impaired neovascularization in response to ischemia [23]. However, the defects in endothelial function and postnatal neovascularization in young CuZnSOD^−/−^ mice are relatively modest, and the effect of aging in these conditions is unknown. Here we studied the response to ischemia in both young and older homozygous CuZnSOD-deficient mice in order to specifically define the role of CuZnSOD and aging for the modulation of neovascularization.

Our results indicate that CuZnSOD expression in hindlimb muscles is decreased with aging. We also demonstrate that CuZnSOD deficiency accelerates vascular aging in the context of tissue ischemia. In fact, young CuZnSOD^−/−^ mice exhibit neovascularisation levels that are similar to that of older control mice, both at the macrovascular (Laser Doppler) and microvascular (vascular density) levels. In older CuZnSOD^−/−^ mice, surgically-induced hindlimb ischemia resulted in severe necrosis and auto-amputation in the second week after surgery (data not shown) and the current study was therefore limited to a one-week follow-up. Although impaired ischemia-induced neovascularisation has previously been shown with aging [5], [6], the current study demonstrates that CuZnSOD deficiency is associated with an important further reduction of blood flow recovery in older animals. At the microvascular level, the negative effect of CuZnSOD deficiency in older animals was mainly seen in arteriolar vessels. Globally, these results suggest that CuZnSOD has an important role in the context of aging to prevent an excessive reduction of neovascularization in response to ischemia.

The mechanisms by which CuZnSOD modulates neovascularization in the context of aging are potentially diverse. Ischemic tissues are characterized by high levels of inflammatory cytokines that can activate ROS production [24]. Excessive production of ROS in pathological conditions leads to cellular toxicity and has been associated with impaired angiogenesis in different animal models [23], [25], [26], [27]. CuZnSOD activity could therefore act as a defense mechanism against excessive oxidative stress accumulation and tissue damage in older animals. Consistent with this, we found that superoxide and ROS production in ischemic tissues were significantly increased by aging and that the highest levels of oxidative stress were found in old CuZnSOD-deficient mice. Increased oxidative stress level in older and CuZnSOD-deficient mice was also associated with higher expression of the stress-induced senescence markers p53 and p16. It is conceivable that high levels of ROS in old CuZnSOD-deficient mice promote endothelial cell dysfunction in ischemic tissues, which would in turn contribute to impair angiogenesis. In addition, excessive ROS production in ischemic tissues could also interfere with the activity of pro-angiogenic factors such as VEGF [25], [26].

In the current study, we propose that another important factor involved in the accelerated vascular aging of CuZnSOD-deficient mice is endothelial progenitor cells (EPCs) dysfunction. The importance of EPCs for the development of neovessels in different physiological and pathological situations has recently been recognized [2], [3], [4]. In healthy individuals or in patients with cardiovascular diseases, aging has been associated with a reduction in the number and/or the functional activities of EPCs [8], [9], [10]. In vivo, young bone marrow-derived EPCs have been shown to restore aging-impaired cardiac angiogenic function [7]. Here we demonstrate that the number of EPCs is significantly and similarly reduced by aging and CuZnSOD deficiency. Our results suggest that CuZnSOD deficiency leads to an age-dependant depletion of EPC reserve in the bone marrow. This could at least in part explain the important reduction of peripheral (spleen) EPCs that we documented in these animals. Future studies are needed to determine whether CuZnSOD deficiency can also impair the mobilization of EPCs from the bone marrow in the context of aging.

Age-dependent endothelial dysfunction has been shown to correlate with impairment in the functional activity of EPCs [28]. Here we found that the functional activities of EPCs are impaired both by aging and CuZnSOD deficiency. However, the effect of aging alone on EPC function (e.g. migration/integration into tubules) was often modest, whereas CuZnSOD deficiency was associated with severe age-dependent defects. This suggests that CuZnSOD could have an important role to limit excessive oxidative stress and preserve EPC angiogenic activities in the context of aging. In fact, EPCs have previously been shown to express higher levels of anti-oxidative enzymes and enhanced protection against oxidative stress compared to mature endothelial cells [29]. Here we found that superoxide levels in EPCs were not significantly increased in older compared to young wild-type animals. However, CuZnSOD deficiency led to an important increase of EPC superoxide levels in older animals. Globally, our results are consistent with a protective role of CuZnSOD against age-dependent increase of oxidative stress levels and impairment of EPC angiogenic activities.

In summary, our study demonstrates that CuZnSOD deficiency leads to accelerated vascular aging and impaired post-natal neovascularization after ischemia. We propose that in the context of aging, CuZnSOD has an essential role to protect against excessive oxidative stress in ischemic tissues and preserve the number and the functional activities of EPCs. The finding that CuZnSOD is essential to maintain EPC functions might have important clinical implications. In ‘healthy’ aging, stable CuZnSOD expression could limit oxidative stress accumulation and help maintaining EPC function and vascular integrity. However, it has been shown that clinical conditions such as diabetes and chronic heart failure are associated with defective levels of antioxidant enzymes, including CuZnSOD [30], [31], [32]. In these patients, aging could lead to accelerated accumulation of ROS, impaired EPC function and defective neovascularization in response to ischemia. CuZnSOD modulation might therefore represent a novel therapeutic avenue to restore EPC functions and neovascularization in aging patients with severe ischemic vascular diseases presenting different cardiovascular risk factors.

## Supporting Information

Figure S1 **CuZnSOD expression in hindlimb muscles of young (Y) and older (O) wild type (CuZnSOD^+/+^) mice (n = 2–4/group).** Results are expressed as density values normalized to Ponceau red staining. Data are mean ± SEM. * *P*<0.05 *vs.* CuZnSOD^+/+^ Y.
(TIF)

Figure S2 **Histo-immunostaining showing expression of p53 (A) and p16 (B) in ischemic hindlimb muscles of young (Y) and older (O) CuZnSOD^+/+^ and CuZnSOD^−/−^ mice.** Representative results are shown.
(TIF)
