# Supplementary figures and images for: Accelerated Vascular Aging in CuZnSOD-Deficient Mice: Impact on EPC Function and Reparative Neovascularization
Source: PLoS One. 2011 Aug 12;6(8):e23308. doi: 10.1371/journal.pone.0023308 (PMC3155535; doi:10.1371/journal.pone.0023308)

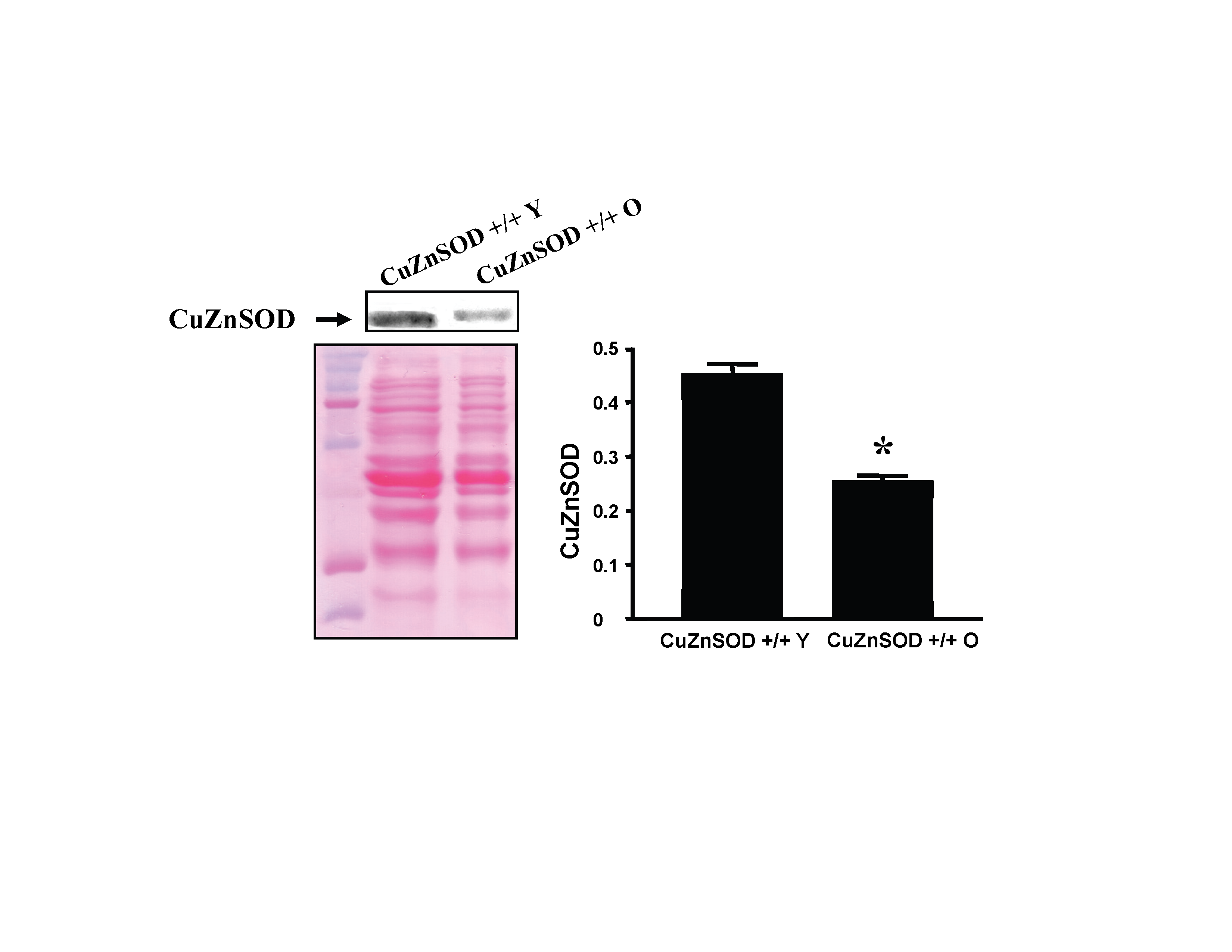

Supplement: Figure S1 — CuZnSOD expression in hindlimb muscles of young (Y) and older (O) wild type (CuZnSOD+/+) mice (n = 2–4/group). Results are expressed as density values normalized to Ponceau red staining. Data are mean ± SEM. * P<0.05 vs. CuZnSOD+/+ Y. (TIF) [file pone.0023308.s001.tif]

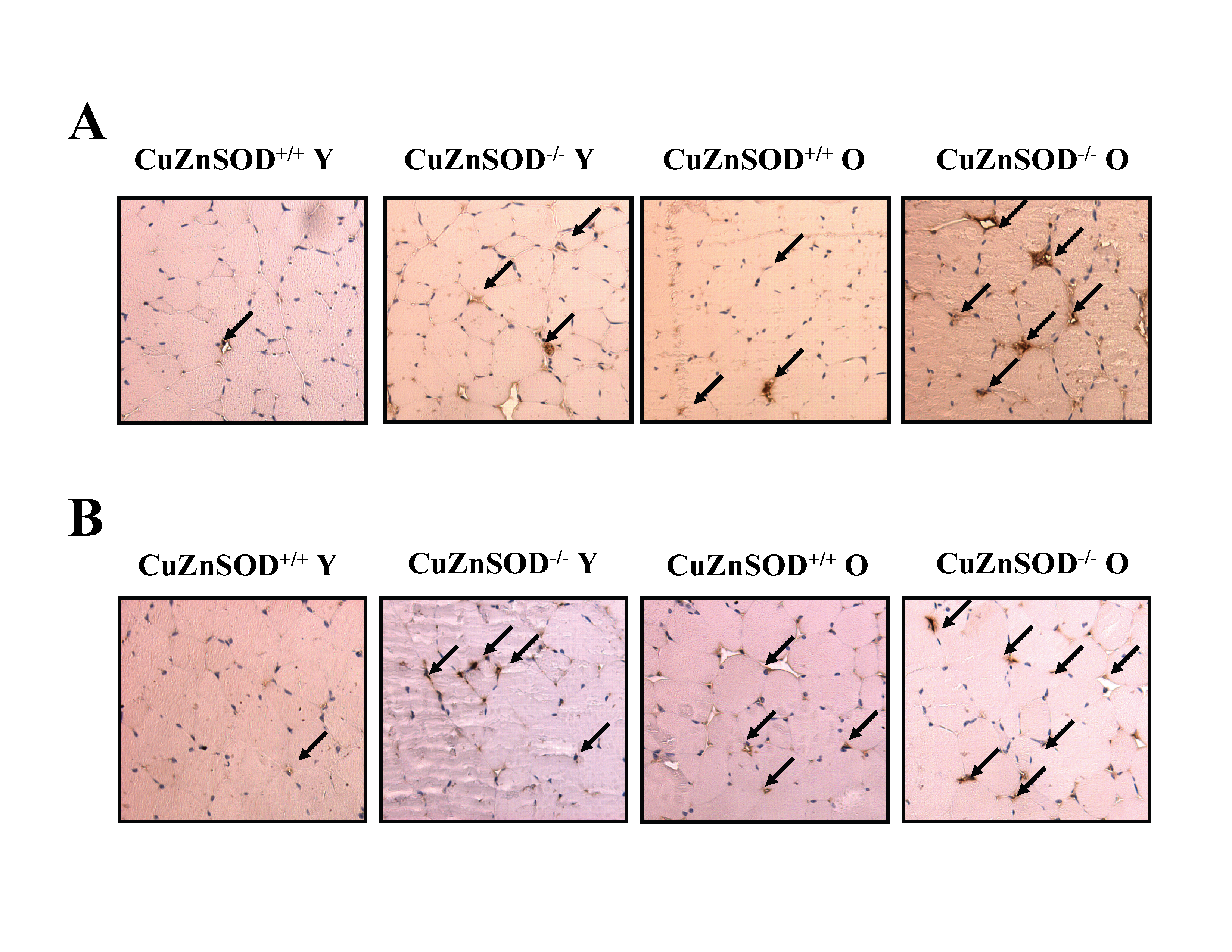

Supplement: Figure S2 — Histo-immunostaining showing expression of p53 (A) and p16 (B) in ischemic hindlimb muscles of young (Y) and older (O) CuZnSOD+/+ and CuZnSOD−/− mice. Representative results are shown. (TIF) [file pone.0023308.s002.tif]
